# Field efficacy of Porcilis® PCV M Hyo versus a licensed commercially available vaccine and placebo in the prevention of PRDC in pigs on a French farm: a randomized controlled trial

**DOI:** 10.1186/s40813-016-0051-0

**Published:** 2017-02-01

**Authors:** Eric Pagot, Martial Rigaut, David Roudaut, Luca Panzavolta, Rika Jolie, Didier Duivon

**Affiliations:** 1 Zoopole Développement-CTPA, Ploufragan, France; 2 MSD Santé Animale, 7, rue Olivier de Serres - Angers Technopole C.S. 17144, 49071 Beaucouzé cedex, France; 3 MSD Animal Health, 2 Giralda Farms, Madison, NJ 07940 USA

**Keywords:** PCV2, *Mycoplasma hyopneumoniae*, Vaccine, Randomised controlled field trial

## Abstract

**Background:**

A controlled, randomised, and blinded trial performed on a conventional French farrow-to-finish farm compared the efficacy of a one-shot bivalent ready to use vaccine, Porcilis® PCV M. Hyo (group PCVM), to that of two commercial vaccines (Ingelvac® Circoflex® + Ingelvac® Mhyo, group ICIM), and to a placebo (group CTL), in preventing the health and economic impacts of Porcine Respiratory Disease Complex (PRDC).

**Material & Methods:**

In this small-scale clinical study, all piglets in each group were administered the vaccine/placebo at weaning age (27 days old). Piglets from either of the three groups were bled at regular intervals from 3 weeks of age until slaughter, in order to assess the infection by the main PRDC infectious agents: *Mycoplasma hyopneumoniae*, PCV2 and PRRSV. Performance, lung checks and slaughter data were collected and analysed.

**Results:**

PCV2 viremia was significantly reduced in both vaccinated groups as compared to the placebo group. Lung lesion score was significantly lower in group PCVM, as compared to groups CTL and ICIM. Average daily weight gain during the finishing period was not significantly different between both vaccinated groups and was significantly higher than in the placebo group (849 g/d in the latter). Carcass results provided a numerical advantage to PCVM group, through improved part of production eligible for premium payment, and superior farmer income; this was a trend and did not reach significance.

**Conclusion:**

The one-shot bivalent vaccine Porcilis® PCV M Hyo proved to be efficacious and convenient to use in a field context of active PCV2 and *M. hyopneumoniae* infections.

## Background and regional context

The term Porcine Respiratory Disease Complex (PRDC) describes pneumonia of multiple, combined causal factors (environmental, management and infectious risk factors play variable roles), resulting in clinical disease and reduced growth over the finishing phase of pig farming (15 to 20 weeks of age) [1]. This multifaceted respiratory condition in fattening pigs can be controlled through the efficient prevention and correction of the relevant risk factors identified by the practitioner of the corresponding farm. In Western France (Brittany), where 60% of the national swine production is concentrated, PRDC components have been extensively studied, regarding both non-infectious [2] and infectious [3] risk factors. The former cross-sectional study evidenced that-under French farming conditions, pneumonia and pleuritis risk factors are mostly different, except for an inappropriate ventilation programme [2]. Regarding infectious risk factors, the respective part of pathogens in PRDC can vary with geographic locations, for instance most of France, with the exception of its Western part, is free of PRRS, while Switzerland is free of *Mycoplasma hyopneumoniae*. For Western France, pathogens found to have a prominent role in PRDC were: *Mycoplasma hyopneumoniae*, Porcine Reproductive and Respiratory Syndrome Virus (PRRSV) and Swine influenza virus (SIV) H1N1, with porcine circovirus type 2 (PCV2) seeming less dominant in the pneumonia-like gross lesions, although it might play a role [2]. Extensive pleuritis lesions were solely found significantly associated with the combination of *Actinobacillus pleuropneumoniae* serotype 2 and PRRSV. In well-performing French farms, the ventilation programme is checked daily by the farmer, and controlled on a monthly basis by trained technicians. The control of infectious risk factors is mostly performed through vaccination (see [4] for a review). However, even though vaccination against enzootic pneumonia is widely implemented, pneumonia-like lesions frequency and detection of *M. hyopneumoniae* remain elevated [5]. Also, it has been assessed that, under Brittany’s farming conditions, there is a significant (*p* < 0.001) negative correlation between pneumonia score and growth [6], with a 0.7% loss of average daily weight gain (ADWG) for each point of pneumonic lung score increase (“Madec” lung scoring is performed on a 1–28 scale) [7]. Farmers are aware of these facts and are therefore eager to challenge the respiratory disease vaccine strategy implemented in their farm in order to benefit from any possible improvement of PRDC control.

Several vaccines against respiratory/systemic infectious agents, which have been evaluated by the European medicines agency (EMA), have recently reached the European market. Among those is, the first ready-to-use bivalent vaccine against PCV2 and *Mycoplasma hyopneumoniae*, Porcilis® PCV M Hyo, with efficacy data published by EMA in its European public assessment report.^1^

The present clinical study provides further evidence of the field efficacy of this vaccine.

## Case farm and trial setting

In December 2014, a conventional 450-sow French farrow-to-finish swine farm with no history of clinical PCV2-associated disease was suggested by its practitioner to our clinical study team as being eligible for a field trial on PCV2 and *Mycoplasma hyopneumoniae* vaccine efficacy. The farm is located in Brittany, in a high-density farming area (Côtes-d’Armor). A pilot survey was implemented to assess the circulation of PCV2 and *M. hyopneumoniae* in the herd.

PCV2 serology (in-house test, Laboratory for Diagnostic Solutions Intervet Boxmeer, the Netherlands) was performed on fifteen piglets of three age classes: 4, 12 and 22 weeks of age. Seropositivity rate was found to be increasing with age, i.e. 28–35%, 38–80% and 67–89% respectively, which was suggestive of an active post-weaning PCV2 circulation (the herd did not vaccinate sows against PCV2).

*M. hyopneumoniae* serology (IDEXX *M. hyo* Ab Test) was performed on fifteen 21-week old pigs, of which 87% were found positive. Although enzootic pneumonia vaccination was routinely performed at weaning on this farm, such high seropositivity rate is strongly suggestive of active *M. hyopneumoniae* infection in the fattening period, on a one-site production system [8]. Furthermore, a lung check was performed at the slaughterhouse in December 2014, which confirmed that 83% of the 23 controlled pigs had pneumonia lesions, a mean lung lesion score (LLS) of 8.4 (‘Madec’ scoring method, on 28, see [6]) and a median LLS of 8.0. Together, these results supported an active *M. hyopneumoniae* infection in the fattening unit.

The PRRS status of the farm had been recently determined by the farm veterinarian, with seropositive sows and pigs becoming infected from 10 weeks of age onwards. PRRSV infection was controlled in the female herd by priming gilts (quarantine) with a modified live vaccine, and boosts at regular intervals with an inactivated vaccine. Serology performed during the trial later confirmed that all piglets seroconverted by 14 weeks of age (data not shown). The same was true for *Haemophilus parasuis* (all pigs seroconverted by week 22), while no seroconversion was measured against *Actinobacillus pleuropneumoniae*, nor SIV (H1N1, H1N2 and H3N2).

Taken together, these results evidenced the presence of PRDC, in compliance with both its clinical expression and the association of pathogens described by Fablet *et al*. in Western France [2]. This farm was among the better half of the farms belonging to the same production cooperative (see Table 1), and the producer accepted his practitioner’s suggestion to add PCV2 valence to the piglets’ vaccination schedule and to compare two options to vaccinate concurrently against *M. hyopneumoniae* and PCV2: a one-shot bivalent ready-to-use (RTU) and a combination of two commercial vaccines applied in two separate injections. This trial was to be carried out according to a controlled, randomised, and blinded design, upon completion of the informed consent form by the farmer.

**Table 1:** Comparative performance results for the trial farm, with reference to the production cooperative it belongs to, and to swine producers in the same region (farm's data are in bold)

	Study farm, average 2015 performance	Average performance of all members of the same cooperative, 2015	Average performance of all producers in Brittany, 2015
Number of pigs produced per sow per year	**28.5**	24.4	23.2
Number of kg liveweight produced per sow per year	**3**,**439**	2,809	2,699
Feed conversion ratio (wean to finish)	**2.80**	2.81	2.80
Mortality rate (includes condemnation at slaughterhouse)	**4.9**%	5.9%	6.2%
Normalised ADWG 8–30 kg liveweight (g/d)	**505**	498	478
Normalised ADWG 30–115 kg liveweight (g/d)	**798**	799	815

Four days before the start of the trial (d-4), 498 healthy suckling piglets within the same farrowing batch were weighed, sexed and individually identified. On d0, these piglets were on average 26.6 days old; they were randomly assigned to one of three treatment groups (see below) taking into account the sex (one randomisation list for each sex), the litter, and the weight (sorted in a random list by sow and weight). They were allocated as they came to hand, until the required number of piglets was reached. In 15 litters, three of the selected piglets (one per treatment group) of comparable girth (corpulence) were further selected for blood sampling and received an additional ear tag, of a different shape and colour. Individual identification of each included piglet allows considering the animal as a statistical unit.

On day 0 (10th of March 2015), all piglets were injected according to their allocated group; blood sampling was also performed. After these procedures, piglets were placed back in their pen of origin, irrespective of their treatment group.

- Group CTL (control): piglets (*n* = 165) were injected in the neck with saline as placebo (2 ml, via the intramuscular route).
- Group PCVM: piglets (*n* = 166) were injected in the neck with the one-shot bivalent RTU vaccine Porcilis® PCV M Hyo (2 ml, via the intramuscular route).
- Group ICIM: piglets (*n* = 167) were injected with both vaccines Ingelvac® CircoFLEX® and Ingelvac® M Hyo, in compliance with the product’s specifications^2^: two injections (1 and 2 ml, respectively) were administered via the IM route on the same side of the neck of the piglets.

All vaccine injections, blood sampling and weighing manipulations of piglets/pigs were performed by trained investigators, from a dedicated intervention team. All other farm routines were performed-without change, by stockmen.

### Assessment of vaccine efficacy

To assess the efficacy of vaccination, the following primary parameters were used: for PCV2 protection, PCV2 viremia (quantitative PCR genomic load); for *M. hyopneumoniae* infection, lung lesion score (LLS) at slaughter; for both pathogens, average daily weight gain (ADWG) over the finishing period (pigs were individually weighed on the farm before departure to slaughter, on the 15th and 16th of July). The secondary efficacy parameters included overall ADWG and mortality (between vaccination and slaughter, including total condemnation).

At inclusion, there were no statistically significant differences in the sex ratio of pigs within each group (*p* = 0.998, Pearson Chi-square), nor in live-weight (*p* = 0.578, least-square means). On this farm, piglets are routinely weaned at 28 days of age, then spend 2 weeks in a nursery room before entering pre-fattening for a 5-week period. At 11 weeks of age, they are transferred to the finishing rooms. At this age, there were no significant differences in average live-weight between groups (*p* = 0.534, analysis of variance). There was, however, a significant difference between groups for the average daily weight gain (ADWG) over that finishing period: PCVM group had a significantly higher ADWG than the CTL group (*p* = 0.011, analysis of variance). These results are detailed in Table 2.

**Table 2:** Numeric data, results, and statistical analysis for each trial group, measures performed over the production period

	Weight at inclusion (kg) (number included)	Weight at transfer to fattening (kg)	Weight before departure (kg)	ADWG, nursery (g/d)	ADWG, fattening (g/d)	Mortality rate incl. condemnations (number of dead pigs)
Group CTL	6.80 (165)	29.56	96.9	438	849.6^a^	2.4% (4)
Group PCVM	6.79 (166)	28.96	99.0	426	880.6^b^	6.0% (9)
Group ICIM	6.66 (167)	29.14	97.9	432	867.2^ab^	3.6% (4)
Total	(498)	NC	NC	NC	NC	NC
*p* value	0.578	0.534	0.193	0.424	0.011	0.218
Test used	Anova	Anova	Anova	Anova	Anova	Chi-square

Values with different letters within the same column are statistically significantly different

*NC* not calculated

**Fig. 1:**
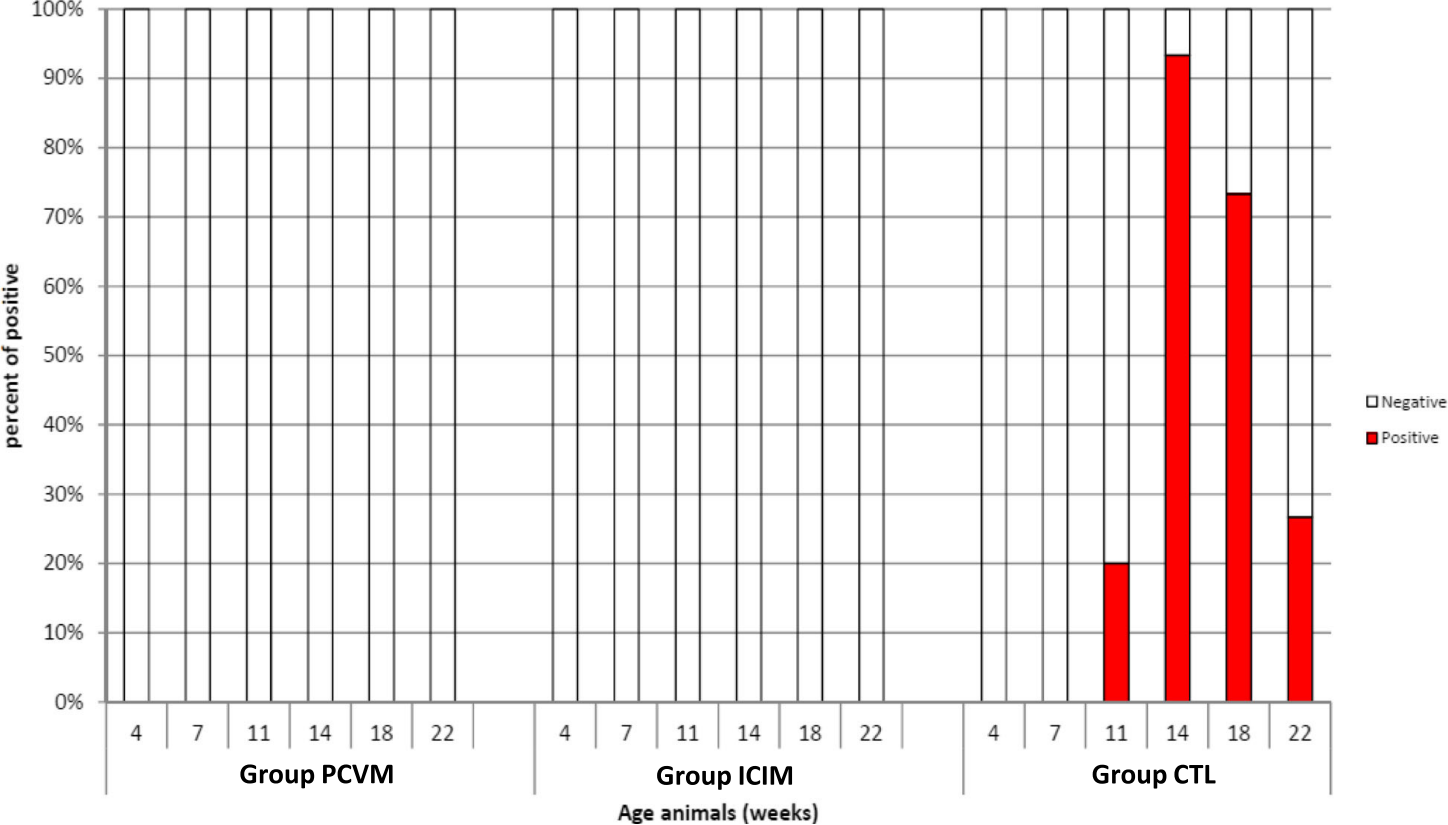
Proportion of piglets with a detectable PCV2 genomic load in serum (qPCR) in each group, across ages

To assess PCV2 viremia, one piglet per litter in each group was bled at regular intervals (4, 7, 11, 14, 18 and 22 weeks of age). Testing was performed at the Laboratory for Diagnostic Solutions Intervet Boxmeer, the Netherlands, with an in-house quantitative PCR (detection limit of 2 log10 copies per μl DNA extract). No PCV2 genome was found in the blood of piglets from PCVM and ICIM groups, at any age sampled. Conversely, in CTL group, PCV2 viremia appeared in 20% of the sampled 11-week old piglets and was detectable in 94% of 14-week old piglets (see Fig. 1). Area Under the Curve (AUC) value was 14.0 (group CTL), 0.2 (group PCVM) and 0.5 (group ICIM). AUC was highly significantly lower in each vaccinated group as compared to the CTL group (in each case, *p* < 0.0001, Anova).

Lung check was performed at the slaughterhouse for a subset of pigs in each group (55 to 69 pigs per group).^3^

**Fig. 2:**
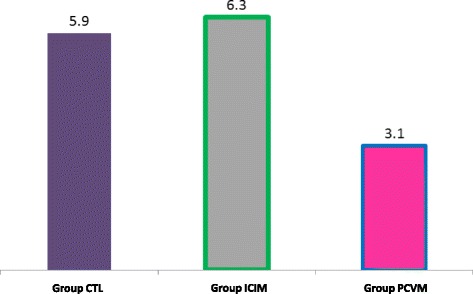
Average lung lesion score (scoring according to Goodwin) in each trial group, in a subset of 179 pigs, from the 498 pigs included

**Fig. 3:**
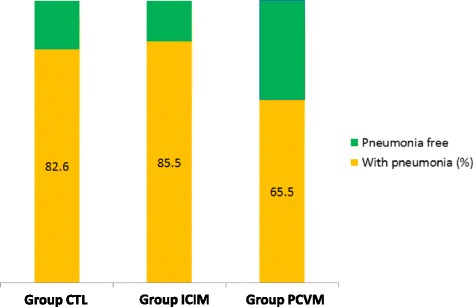
Percentage of pigs with pneumonia, in each trial group, in a subset of 179 pigs, from the 498 pigs included

Lung scoring was performed individually according to the method established by Goodwin [9]. The mean lung lesion score of group PCVM was found to be lower, with a significant difference (*p* = 0.007, Kruskal-Wallis test, see Fig. 2), compared with the 2 other groups. Also, the proportion of lungs with pneumonic lesions was significantly higher in both group CTL and group ICIM, as compared to group PCVM (*p* = 0.021, Pearson Chi-square, see Fig. 3). Taken together, these results are in favour of the use of multivalent vaccines/vaccine programmes in prevention of PRDC. Under the condition that the selected valences are adapted to the local combination of the pathogens implied in PRDC, this trial adds-up to the growing body of evidence of the efficacy of this intervention, as has recently been demonstrated in Germany [10] against PCV2 and *M. hyopneumoniae* or Korea [11] against PCV2 and PRRSV, with a more limited number of primary parameters.

Slaughter data were sent by the slaughterhouse to the farmer under an electronic format (Microsoft Excel® tabs) and were compiled with the farmer’s agreement. These complete data provided secondary parameters: mortality rate (which includes full carcass condemnation at the slaughterhouse), average age at slaughter, average net carcass weight (excluding the condemned pieces), average amount of quality premiums for which the farmer was eligible and average per-pig income (calculation includes vaccination costs). These data are detailed in Table 3. Statistical significance was not reached between any groups for any of these parameters. There was however a trend for improved revenue paid to the farmer in group PCVM (*p* = 0.08), through a larger number of pigs eligible f﻿or a premium (*p* = 0.07). A larger number of pigs in the trial may have resulted in statistical significance. This latter observation is in parallel with the recent assessment of the antimicrobial metaphylaxis in fattening pigs affected by PRDC in PRRS-free herds [12], where growth retardation was only compensated in the lighter pigs in the studied cohort, i.e. where the intervention has limited positive impact in terms of animal health as well as of financial benefit.

**Table 3:** Parameters measured at slaughterhouse, for a subset of each treatment group

	Number of lung checks	Average lung score (on 55)	Frequency of pneumonic lungs (%)	Age at slaughter (d)	Net carcass weight (kg)	Quality premium (€ cent per kg)	Income per pig^a^ (€/head)
Group CTL	69	5.9^a^	82.6^a^	187.1	90.6	13.5	139.6
Group PCVM	55	3.1^b^	65.5^b^	184.5	91.6	14.1	141.6
Group ICIM	55	6.3^a^	85.5^a^	185.6	91.0	12.3	139.0
*p* value	NC	0.007	0.021	0.162	0.246	0.071	0.082
Test used	NA	Kruskal-Wallis	Pearson Chi-2	Kruskal-Wallis	Anova	Anova	Anova

Values with different letters within the same column present a statistically significant difference

^a^Vaccination cost included

*NC* not calculated

*NA* not applicable

## Conclusion

Pigs in the control group presented an elevated PCV2 viremia between 11 and 22 weeks of age. Upon slaughter, more than 80% of them presented *M. hyopneumoniae*-like lung lesions, with average LLS of 5.9. These elements confirm that both PRDC pathogens were actively circulating in the trial farm. A highly significant prevention of PCV2 viremia was observed in both vaccinated groups.

Compared to the group ICIM, the group PCVM had significantly better outcome regarding the frequency of pneumonia (−20%) and LLS (3.1 vs. 6.3). Furthermore, the group PCVM also produced an improved ADWG in fattening (+13 g/d), carcasses quality premium (+1,8 € cts/kg) and average per-pig income (+2,6 €); these favorable results neared significance. In this field trial, Porcilis® PCV M Hyo proved to be efficacious in protecting piglets against both PCV2 viremia and the impact of *M. hyopneumoniae* infection, in a context of active PRDC and PCV2 infection.

## Abbreviations

ADWG: Average daily weight gain
AUC: Area under the curve
LLS: Lung lesion score
PCR: Polymerase chain reaction
PCV2: Porcine Circovirus type 2
PRDC: Porcine respiratory disease complex
PRRS: porcine reproductive and respiratory syndrome
PRRSV: Porcine reproductive and respiratory syndrome virus
RTU: Ready-to-use
SIV: Swine influenza virus

## Footnotes

1 This EPAR is freely available online on the website of EMA: http://www.ema.europa.eu/ema/index.jsp?curl=pages/medicines/veterinary/medicines/003796/vet_med_000307.jsp&mid=WC0b01ac058008d7a8.

2 For CircoFLEX®, see at http://www.ema.europa.eu/docs/en_GB/document_library/EPAR_-_Product_Information/veterinary/000126/WC500062388.pdf. For Ingelvac® M. Hyo, see French SPCs at http://www.ircp.anmv.anses.fr/rcp.aspx?NomMedicament=INGELVAC+M.HYO

3 Due to social contemporary social disruption at the time of the trial, slaughterhouse of destination of some batches has been changed at the last minute, without the lung check personnel being warned. Some animals have been missed, in each trial group.
